# Comprehensive Genomic Profiling of Rare Tumors: Routes to Targeted Therapies

**DOI:** 10.3389/fonc.2020.00536

**Published:** 2020-04-21

**Authors:** Shuhang Wang, Rongrong Chen, Yu Tang, Yue Yu, Yuan Fang, Huiyao Huang, Dawei Wu, Hong Fang, Ying Bai, Chao Sun, Anqi Yu, Qi Fan, Dejian Gu, Xin Yi, Ning Li

**Affiliations:** ^1^Clinical Cancer Center, National Cancer Center/National Clinical Research Center for Cancer/Cancer Hospital, Chinese Academy of Medical Sciences and Peking Union Medical College, Beijing, China; ^2^Department of Medical Center, Geneplus-Beijing Institute, Beijing, China

**Keywords:** rare tumors, genomic profile, targetable genomic alterations, actionable mutation, NGS, China

## Abstract

Comprehensive Genomic Profiling may be informative for novel treatment strategies and to improve outcomes for patients with rare tumors. This study aims to discover opportunities for use of targeted therapies already approved for routine use in patients with rare tumors. Solid tumors with an incidence lower than 2.5/100,000 per year was defined as rare tumors in China after comprehensive analysis based on epidemiological data and current availability of standardized treatment. Genomic data of rare tumors from the public database cBioPortal were compared with that of the Chinese population for targetable genomic alterations (TGAs). TGAs were defined as mutations of *ALK, ATM, BRAF, BRCA1, BRCA2, CDKN2A, EGFR, ERBB2, FGFR1,2,3, KIT, MET, NF1, NTRK1,2,3, PIK3CA, PTEN, RET*, and *ROS1* with level 1 to 4 of evidence according to the OncoKB knowledge database. Genomic data of 4,901 patients covering 63 subtypes of rare tumor from cBioPortal were used as the western cohort. The Chinese cohort was comprised of next generation sequencing (NGS) data of 1,312 patients from across China covering 67 subtypes. Forty-one subtypes were common between the two cohorts. The accumulative prevalence of TGAs was 20.40% (1000/4901) in cBioPortal cohort, and 53.43% (701/1312) in Chinese cohort (*p* < 0.001). Among those 41 overlapping subtypes, it was still significantly higher in Chinese cohort compared with cBioPortal cohort (54.1%% vs. 26.1%, *p* < 0.001). Generally, targetable mutations in *BRAF, BRCA2, CDKN2A, EGFR, ERBB2, KIT, MET, NF1, ROS1* were ≥3 times more frequent in Chinese cohort compared with that of the cBioPortal cohort. Cancer of unknown primary tumor type, gastrointestinal stromal tumor, gallbladder cancer, intrahepatic cholangiocarcinoma, and sarcomatoid carcinoma of the lung were the top 5 tumor types with the highest number of TGAs per tumor. The incidence of TGAs in rare tumors was substantial worldwide and was even higher in our Chinese rare tumor population. Comprehensive genomic profiling may offer novel treatment paradigms to address the limited options for patients with rare tumors.

## Introduction

Molecular profiling to identify potential therapeutic targets has been widely applied in common tumors such as lung cancer (1, 2), breast cancer (3, 4), melanoma (5), and colorectal cancer (6, 7). The use of targeted therapy in selected patients can significantly improve outcomes. Increasingly, clinical trials feature targeted therapeutic agents or require a specific biomarker for entry (8, 9). However, limited information is available regarding the utility of targeted therapy for rare tumors (10, 11). What's more, while rare individually, rare tumors cumulatively account for over 20% of adult malignant neoplasms in the United States (12, 13).

There is no universally applied definition for rare tumors (Table 1). The European Society for Medical Oncology (ESMO) defines a rare tumor as a tumor with an annual incidence of 6/10,000 (14) in Europe. The National Cancer Institute (NCI) (https://www.cancer.gov/publications/dictionaries/cancer-terms/def/791790) and Food and Drug Administration (FDA) (http://www.ncbi.nlm.nih.gov/pmc/articles/PMC2789814/) defines it as a tumor with an annual incidence of <15/10,000 in the US. According to the NCI definition, lung cancer, colon cancer, breast cancer, prostate cancer, endometrial carcinoma, rectal cancer, ovarian cancer, kidney cancer, melanoma, non-Hodgkin lymphoma, and gastric cancer belong to common cancers.

**Table 1:** Worldwide rare tumor prevalence.

**Source**	**Type**	**Definition**	**Link for information**
FDA	Rare disease	<200,000 in US	https://www.fda.gov/industry/developing-products-rare-diseases-conditions
	Rare tumor	<15/100,000 per year	http://www.ncbi.nlm.nih.gov/pmc/articles/PMC2789814/
NCI	Rare cancer	<15/100,000 per year	https://www.cancer.gov/publications/dictionaries/cancer-terms/def/791790
EMA	Rare disease	<1/2000	http://www.eurordis.org/about-rare-diseases
	Rare tumor	<6/100,000 per year	http://www.rarecancerseurope.org/About-Rare-Cancers

There is some discordance between these definitions and data specific to China. While esophageal cancer and hepatocellular carcinoma are rare tumors according to NCI definition, these are common in China based on annual incidence. On the other hand, skin tumors, especially basal cell carcinoma, are common tumors in the United States, with an incidence of 255.6/100,000 (15), but are relatively rare in China (14) (2.4/100,000 for all skin tumors). This suggests that the definitions from US and Europe were possibly not appropriate in China based on the different incidences and prevalence of tumors.

This study analyzed data from the National Cancer Registry office of the National Cancer Center (16) and integrated it with presently available treatment options to generate a definition of rare tumors specific to China. Subsequently, available data for targetable genomic alterations (TGAs) of two cohorts of rare tumors from the cBioPortal and Geneplus databases were collected and analyzed. Our work provides valuable knowledge to guide personalized, targeted therapy for rare tumors.

## Methods

### Definition of Rare Tumors in China

We consulted National Cancer Registry of the National Cancer Center, China (16) and generated an estimation of incidence of tumors in mainland China. Tumor types were classified according to the International Classification of Diseases (ICD), and we comprehensively synthesized the epidemiology data and availability of standard treatment in China as well as opinions of experts from National Cancer Center. We then defined rare tumors in China according to the following standardizations (Table 2):

1. First, we eliminated the tumors from systems or organs which have consensus or guidelines for treatment in China; an incidence of “2.5/100,000 per year” was selected as a cut-off value for “rare tumor” for tumors with unique ICD codes listed with systems or organs;
2. Secondly, we searched OncoTrees (http://oncotree.mskcc.org/) to further investigate the subtypes of those common tumors that (1) have a distinct ICD code and (2) exhibit an incidence >2.5/100,000 per year in China. We included subtypes of those tumors after further confirming that the incidence of which was ≤2.5/100,000 per year in China by searching Pubmed database (https://www.ncbi.nlm.nih.gov/pubmed/) and the China National Knowledge Infrastructure (CNKI) database;
3. Finally, we also included cancers of unknown primary (CUP) tumors, not only because the incidence of those tumors was ≤2.5/100,000 per year in China, but also because there were no consensus or guidelines for treatment of CUP in China.

**Table 2:** Rare tumors with limited therapeutic strategy in China.

**System**	**ICD**	**Site**	**Tumors subtypes**
Digestive	C24	Biliary tract	Perihilar cholangiocarcinoma
Digestive	C24	Biliary tract	Extrahepatic cholangiocarcinoma
Digestive	C24	Biliary tract	Intrahepatic cholangiocarcinoma
Digestive	C24	Biliary tract	Pancreatobiliary ampullary carcinoma
Digestive	C23	Gallbladder	Gallbladder cancer
Digestive	C17	Small bowel	Small bowel well-differentiated neuroendocrine tumor
Digestive	C17	Small bowel	Duodenal adenocarcinoma
Digestive	C17	Small bowel	Small intestinal carcinoma
Digestive	C22	Liver	Hepatoblastoma
Digestive	C22	Liver	Liver Angiosarcoma
Digestive	C25	Pancreas	Pancreatoblastoma
Digestive	C18	Colon	Medullary Carcinoma Of The Colon
Endocrine	C74	Adrenal	Adrenocortical carcinoma
Endocrine	C75	Pituitary	Pituitary carcinoma
Endocrine	C73	Thyroid	Medullary thyroid cancer
Neural system	C72, C70	Brain	Anaplastic astrocytoma
Neural system	C72, C70	Brain	Anaplastic oligodendroglioma
Neural system	C72, C70	Brain	Anaplastic oligoastrocytoma
Neural system	C72, C70	Brain	Glioblastoma
Neural system	C72, C70	Brain	Astrocytoma
Neural system	C72, C70	Brain	Diffuse intrinsic pontine glioma
Neural system	C72, C70	Brain	Oligodendroglioma
Neural system	C72, C70	Brain	Oligoastrocytoma
Neural system	C72, C70	Brain	High-grade glioma(NOS)
Neural system	C72, C70	Brain	Primitive neuroectodermal tumor
Neural system	C72, C70	Brain	Olfactory neuroblastoma
Neural system	C72, C70	Brain	Medulloepithelioma
Neural system	C72, C70	Brain	Medulloblastoma
Neural system	C72, C70	Brain	Medullomyoblastoma
Neural system	C72, C70	Brain	Ganglioneuroblastoma
Neural system	C72, C70	Brain	Melanotic medulloblastoma
Neural system	C72, C70	Brain	Medulloblastoma with extensive nodularity
Neural system	C72, C70	Brain	Embryonal tumor with abundant neuropil and true rosettes
Neural system	C72, C70	Brain	Atypical teratoid/rhabdoid tumor
Neural system	C72, C70	Brain	Large cell/anaplastic medulloblastoma
Neural system	C72, C70	Brain	Desmoplastic/nodular medulloblastoma
Neural system	C72, C70	Brain	Neuroblastoma
Neural system	C72, C70	Brain	Hemangioblastoma
Neural system	C72, C70	Brain	Mesenchymal chondrosarcoma of the CNS
Neural system	C72, C70	Brain	Papillary meningioma
Neural system	C72, C70	Brain	Atypical meningioma
Neural system	C72, C70	Brain	Anaplastic meningioma
Neural system	C72, C70	Brain	Clear cell meningioma
Neural system	C72, C70	Brain	Meningioma
Neural system	C72, C70	Brain	Chordoid meningioma
Neural system	C72, C70	Brain	Rhabdoid meningioma
Neural system	C72, C70	Brain	Malignant teratoma
Neural system	C72, C70	Brain	Embryonal Carcinoma
Neural system	C72, C70	Brain	Choriocarcinoma
Neural system	C72, C70	Brain	Astroblastoma
Neural system	C72, C70	Brain	Ependymoma
Neural system	C72, C70	Brain	Anaplastic ependymoma
Neural system	C47	Peripheral nerve	Malignant peripheral nerve sheath tumor
Reproductive	C60	Penile	Penile squamous cell carcinoma
Reproductive	C52, C51	Vulva/vagina	Squamous cell carcinoma of the vulva/vagina
Reproductive	C52, C51	Vulva/vagina	Vaginal adenocarcinoma
Reproductive	C52, C51	Vulva/vagina	Mucinous adenocarcinoma of the vulva/vagina
Reproductive	C52, C51	Vulva/vagina	Poorly differentiated vaginal carcinoma
Reproductive	C52, C51	Vulva/vagina	Germ cell tumor of the vulva
Reproductive	C61	Prostate	Prostate small cell carcinoma
Reproductive	C54	Uterus	Uterine adenosarcoma
Reproductive	C54	Uterus	Endometrial stromal sarcoma
Reproductive	C56	Ovary	Dysgerminoma
Reproductive	C56	Ovary	Ovarian carcinosarcoma/malignant mixed mesodermal tumor
Reproductive	C56	Ovary	Brenner tumor, malignant
Reproductive	C56	Ovary	Clear cell ovarian cancer
Reproductive	C56	Ovary	Endometrioid ovarian cancer
Reproductive	C56	Ovary/vulva/vagina/brain/testis,	Embryonal carcinoma
Soft tissue	C49	Soft tissue	Desmoplastic small-round-cell tumor
Soft tissue	C49	Soft tissue	Low-grade fibromyxoid sarcoma
Soft tissue	C49	Soft tissue	Rhabdomyosarcoma
Soft tissue	C49	Soft tissue	Synovial sarcoma
Soft tissue	C49	Soft tissue	Myofibroma
Soft tissue	C49	Soft tissue	Myopericytoma
Soft tissue	C49	Soft tissue	Myxofibrosarcoma
Soft tissue	C49	Soft tissue	Leiomyosarcoma
Soft tissue	C49	Soft tissue	Aggressive angiomyxoma
Soft tissue	C49	Soft tissue	Soft tissue myoepithelial carcinoma
Soft tissue	C49	Soft tissue	Alveolar soft part sarcoma
Soft tissue	C49	Soft tissue	Epithelioid sarcoma
Soft tissue	C49	Soft tissue	Epithelioid hemangioendothelioma
Soft tissue	C49	Soft tissue	Dendritic cell sarcoma
Soft tissue	C49	Soft tissue	Clear cell sarcoma
Soft tissue	C49	Soft tissue	Undifferentiated pleomorphic sarcoma/malignant fibrous histiocytoma/high-grade spindle cell sarcoma
Soft tissue	C49	Soft tissue	Gastrointestinal stromal tumor
Soft tissue	C49	Soft tissue	Sarcoma (NOS)
Soft tissue	C49	Soft tissue	Fibrosarcoma
Soft tissue	C49	Soft tissue	Hemangioma
Soft tissue	C49	Soft tissue	Intimal sarcoma
Soft tissue	C49	Soft tissue	Glomangiosarcoma
Soft tissue	C49	Soft tissue	Angiosarcoma
Soft tissue	C49	Soft tissue	Inflammatory myofibroblastic tumor
Soft tissue	C49	Soft tissue	Desmoid/aggressive fibromatosis
Soft tissue	C49	Soft tissue	Liposarcoma
Bone	C40, C41	Bone	Chondrosarcoma
Bone	C40, C41	Bone	Chordoma
Bone	C40, C41	Bone	Osteosarcoma
Bone	C40, C41	Bone	Ewing sarcoma
Skin	C44	Skin	Basal cell carcinoma
Skin	C44	Skin	Dermatofibrosarcoma protuberans
Skin	C44	Skin	Merkel cell carcinoma
Skin	C44	Skin	Cutaneous Squamous Cell Carcinoma
Skin	C44	Skin	Aggressive digital papillary adenocarcinoma
Skin	C44	Skin	Sebaceous carcinoma
Skin	C44	Skin	Skin adnexal carcinoma
Skin	C44	Skin	Sweat gland adenocarcinoma
Skin	C44	Skin	Sweat gland carcinoma/apocrine eccrine carcinoma
Lung	C39	Lung	Mucoepidermoid carcinoma of the lung
Lung	C39	Lung	Spindle cell carcinoma of the lung
Lung	C39	Lung	Lymphoepithelioma-like carcinoma of the lung
Lung	C39	Lung	Giant cell carcinoma of the lung
Lung	C39	Lung	Basaloid large cell carcinoma of the lung
Lung	C39	Lung	Clear cell carcinoma of the lung
Lung	C39	Lung	Adenoid cystic carcinoma of the lung
Lung	C39	Lung	Mucoepidermoid carcinoma of the lung
Lung	C39	Lung	Sarcomatoid carcinoma of the lung
Breast	C50	Breast	Breast invasive carcinosarcoma
Breast	C50	Breast	Adenoid cystic breast cancer
Breast	C50	Breast	Breast carcinoma with signet ring
Breast	C50	Breast	Breast invasive mixed mucinous carcinoma
Urinary	C67	Bladder	Plasmacytoid/signet ring cell bladder carcinoma
Urinary	C67	Bladder	Sarcomatoid carcinoma of the urinary bladder
Urinary	C67	Bladder	Small cell bladder cancer
Urinary	C64	Kidney	Renal non-clear cell carcinoma
Others	C45, C48	Pleura, Peritonea	Pleural mesothelioma
Others	C45, C48	Pleura, Peritonea	Pleuropulmonary blastoma
Others	C45, C48	Pleura, peritonea	Peritoneal mesothelioma
Others	C38	Heart	Primary heart malignant tumor
Others	C37	Thymus	Thymic carcinoma
Others	C06	Head and neck	Acinic cell carcinoma
Others	C06	Head and neck	Adenoid cystic carcinoma
Others	C06	Head and neck	Mammary analogue secretory carcinoma of salivary gland origin
Others	C06	Head and neck	Mucoepidermoid carcinoma
Others	C06	Head and neck	Myoepithelial carcinoma
Others	C06	Head and neck	Salivary adenocarcinoma
Others	C06	Head and neck	Salivary duct carcinoma
Others	C06	Head and neck	Epithelial-myoepithelial carcinoma
Others	C06	Head and neck	Clear cell odontogenic carcinoma
Others	C06	Head and neck	Sinonasal adenocarcinoma
Others	C06	Head and neck	Sinonasal undifferentiated carcinoma
Others	C80, C76	Unknown	Cancer of unknown primary

### Definition of Targetable Mutations According to the OncoKB Framework

The actionabilities of genetic alterations were mainly based on the OncoKB knowledge database (https://oncokb.org). Utilizing the OncoKB framework, mutations could be classified into 4 main levels of evidence for biomarker-guided therapy and those with unknown significance. OncoKB is a precision oncology knowledge base and contains information about the effects and treatment implications of specific cancer gene alterations. It is developed and maintained by the Knowledge Systems group in the Marie Josée and Henry R. Kravis Center for Molecular Oncology at Memorial Sloan Kettering Cancer Center (MSK) (17). Curated by a network of clinical fellows, research fellows, and faculty members at MSK, OncoKB contains detailed information about specific alterations in 668 cancer genes. The information is compiled from various sources, such as guidelines from the FDA, NCCN, or ASCO, ClinicalTrials.gov and the scientific literature. Level 1 is an FDA-recognized biomarker predictive of response to an FDA-approved drug in this indication. Level 2 is standard care biomarker predictive of response to an FDA-approved drug in this indication (2A) or in another indication, but not standard care in this indication (2B). Level 3 is compelling clinical evidence supports the biomarker as being predictive of response to a drug in this indication (3A) or in another indication (3B). Level 4 is compelling biological evidence supports the biomarker as being predictive of response to a drug (Supplementary Table 1).

### cBioPortal

The cBioPortal for Cancer Genomics was originally developed at Memorial Sloan Kettering Cancer Center. The public cBioPortal site is hosted by the Center for Molecular Oncology at MSK. The cBioPortal currently hosts more than 40 datasets, including TCGA and other large-scale genomic studies, and makes them available for bulk download. Data from OCG's TARGET Initiative will be added to the database in the next year. The data types from the 13,000+ tumor samples include mutations, copy number alterations, mRNA expression changes, and DNA methylation values, as well as clinical parameters, such as disease-free survival.

(https://www.cbioportal.org/datasets).

### Estimation of Targetable Mutations

To estimate the prevalence of targetable mutations in rare tumors, we queried the cBioPortal database using the genes listed in Supplementary Table 1 in a manually curated set of 175 non-redundant studies, including TCGA and non-TCGA studies, with no overlapping samples. Mutations of those genes were downloaded and filtered with the annotated oncoKB levels of evidence. Only mutations of level 1-4 were kept for further analysis. To calculate the prevalence, the cumulative number of targetable mutations in each cancer was divided by total numbers of samples for that cancer. The same criteria and workflow were used for the Chinese patient cohort.

### Patient Recruitment

We retrospectively analyzed genomic profiling data of 1,312 patients with rare tumors from Geneplus database. This database contained patients enrolled from multiple hospitals of China from September 2015 to October 2019 (18, 19). All patients received next-generation sequencing (NGS) testing in Geneplus-Beijing Institute after obtaining written informed consent. Meanwhile, all the patients were stratified into different clinicopathological subgroups according to OncoTree system (http://oncotree.mskcc.org/).

All tissues samples included in this study underwent an onsite pathology review to confirm histologic classification and tumor tissue adequacy, which required a minimum of 20% of tumor cells. Genomic profiling was performed in a College of American Pathologists–accredited laboratory (Geneplus-Beijing) using the Illumina Nextseq CN 500 or Gene+Seq 2000 instrument (20, 21). Briefly, serial sections from formalin-fixed paraffin-embedded (FFPE) tumor tissues were used for genomic tumor DNA extraction using the QIAamp DNA mini kit (Qiagen, Valencia, CA). ctDNA was isolated from 4 to 5 mL of plasma using the QIAamp Circulating Nucleic Acid Kit (Qiagen, Valencia, CA). DNA from leukocytes was extracted using the DNeasy Blood Kit (Qiagen, Valencia, CA). Sequencing libraries were prepared from ctDNA using KAPA DNA Library Preparation Kits (Kapa Biosystems, Wilmington, MA, USA), and genomic DNA sequencing libraries were prepared with Illumina TruSeq DNA Library Preparation Kits (Illumina, San Diego, CA). Libraries were hybridized to custom-designed biotinylated oligonucleotide probes (Roche NimbleGen, Madison, WI, USA) targeting 59-1021 genes (~1.4 Mbp genomic regions of 1021 cancer-related genes or ~230 Kbp genomic regions of 59 genes) (Supplementary Tables 2, 3). Prepared libraries were sequenced on using the Illumina Nextseq CN 500 (Illumina, San Diego, CA) or Gene+Seq 2000 (Geneplus-Beijing, China).

Sequencing data were analyzed using default parameters. Adaptor sequences and low-quality reads were removed. The clean reads were aligned to the reference human genome (hg19) using Burrows-Wheeler Aligner (BWA; version 0.7.12-r1039). Realignment and recalibration were performed using GATK (version 3.4-46-gbc02625). Single nucleotide variants (SNV) were called using MuTect (version 1.1.4) and NChot, a software developed in-house to review hotspot variants (22). Small insertions and deletions (InDels) were determined by GATK. Somatic copy number alterations were identified with CONTRA (v2.0.8). The final candidate variants were all manually verified using Integrative Genomics Viewer.

Targeted capture sequencing required a minimal mean effective depth of coverage of 300× in tissues and 1,000× in plasma samples. For the 1,312 patients included in our study, the mean effective depth of coverage is 1,295× in tissues and 2,014× in plasma samples and 299× in germline DNA samples (Supplementary Table 4).

TGAs simultaneously detected by this assay included base substitutions, short insertions and deletions, focal gene amplifications and homozygous deletions (copy number alterations) and select gene fusions and rearrangements. Variants were filtered to exclude synonymous variants, known germline variants in dbSNP, and variants that occur at a population frequency of >1% in the Exome Sequencing Project. Germline variants were interpreted following ACMG guidelines, and the variants were classified as pathogenic, likely pathogenic, unknown significance, likely benign, and benign.

### Statistics

The Chi-square test or Fisher's exact test was performed to compare frequency targetable mutations between groups. All statistical analysis was performed with SPSS (v.23.0; STATA, College Station, TX, USA) or GraphPad Prism (v. 6.0; GraphPad Software, La Jolla, CA, USA) software. Statistical significance was defined as a two-sided *P*-value of < 0.05.

## Results

### Mutation Profiling of Rare Tumors in cBioPortal Database

Rare tumors according to our China-specific definition included 141 tumor types. We analyzed a total of 45,666 samples from the cBioPortal database and identified 4,901 samples of rare tumors that matched our definition, representing 63 of the 141 possible tumor types. Neuroblastoma, adenoid cystic carcinoma, Ewing sarcoma, astrocytoma, and oligodendroglioma were the top 5 rare tumors, with 1321, 323, 263, 250, and 229 samples, respectively. One thousand (20.4%, 1000/4901) targetable mutations were identified in the 4901 samples, with *PIK3CA, PTEN, KIT, CDKN2A, ATM, FGFR, BRAF, NF1, ALK*, and *BRCA2* as the top 10 genes with targetable mutations identified in 266, 149, 119, 112, 75, 66, 33, 33, 27, and 27 samples, respectively (Table 3 and Supplementary Table 5).

**Table 3:** Prevalence of targetable mutations in rare tumor samples from cBioPortal database.

**System**	**ICD**	**Site**	**Tumors, including but not restricted to**	**All cases**	**Cases with targetable mutations**	**Prevalence of targetable mutations # (%)**
Digestive	C24	Biliary tract	Perihilar cholangiocarcinoma	5	1	20.0
Digestive	C24	Biliary tract	Extrahepatic cholangiocarcinoma	27	7	25.9
Digestive	C24	Biliary tract	Intrahepatic cholangiocarcinoma	186	75	40.3
Digestive	C24	Biliary tract	Pancreatobiliary ampullary carcinoma	9	5	55.6
Digestive	C23	Gallbladder	Gallbladder cancer	81	20	24.7
Digestive	C17	Small bowel	Duodenal adenocarcinoma	3	6	200.0
Digestive	C18	Colon	Medullary carcinoma of the colon	1	5	500.0
Endocrine	C74	Adrenal	adrenocortical carcinoma	118	8	6.8
Endocrine	C73	Thyroid	Medullary thyroid Cancer	17	12	70.6
Neural system	C72, C70	Brain	Anaplastic astrocytoma	110	36	32.7
Neural system	C72, C70	Brain	Anaplastic oligodendroglioma	52	20	38.5
Neural system	C72, C70	Brain	Glioblastoma	15	17	113.3
Neural system	C72, C70	Brain	Astrocytoma	250	46	18.4
Neural system	C72, C70	Brain	Diffuse intrinsic pontine glioma	3	1	33.3
Neural system	C72, C70	Brain	Oligodendroglioma	229	41	17.9
Neural system	C72, C70	Brain	Oligoastrocytoma	147	18	12.2
Neural system	C72, C70	Brain	Primitive neuroectodermal tumor	2	1	50.0
Neural system	C72, C70	Brain	Medulloblastoma	166	8	4.8
Neural system	C72, C70	Brain	Neuroblastoma	1,321	27	2.0
Neural system	C72, C70	Brain	Embryonal carcinoma	36	1	2.8
Neural system	C72, C70	Brain	Choriocarcinoma	11	1	9.1
Neural system	C72, C70	Brain	Ependymoma	11	1	9.1
Neural system	C72, C70	Brain	Anaplastic ependymoma	7	2	28.6
Neural system	C47	Peripheral nerve	Malignant peripheral nerve sheath tumor	35	5	14.3
Reproductive	C60	Penile	Penile squamous cell carcinoma	6	5	83.3
Reproductive	C52, C51	Vulva/vagina	Squamous cell carcinoma of the vulva/vagina	19	7	36.8
Reproductive	C61	Prostate	Prostate small cell carcinoma	7	6	85.7
Reproductive	C56	Ovary	Ovarian carcinosarcoma/malignant mixed mesodermal tumor	12	3	25.0
Reproductive	C56	Ovary	Endometrioid ovarian cancer	7	8	114.3
Reproductive	C56	Ovary/vulva/vagina/brain/testis,	Embryonal carcinoma	36	1	2.8
Soft tissue	C49	Soft tissue	Rhabdomyosarcoma	54	6	11.1
Soft tissue	C49	Soft tissue	Synovial sarcoma	44	3	6.8
Soft tissue	C49	Soft tissue	Myxofibrosarcoma	32	4	12.5
Soft tissue	C49	Soft tissue	Leiomyosarcoma	142	19	13.4
Soft tissue	C49	Soft tissue	Soft tissue myoepithelial carcinoma	6	2	33.3
Soft tissue	C49	Soft tissue	Undifferentiated pleomorphic sarcoma/malignant fibrous histiocytoma/high-grade spindle cell sarcoma	109	21	19.3
Soft tissue	C49	Soft tissue	Gastrointestinal stromal tumor	137	119	86.9
Soft tissue	C49	Soft tissue	Fibrosarcoma	5	0	0.0
Soft tissue	C49	Soft tissue	Angiosarcoma	84	24	28.6
Soft tissue	C49	Soft tissue	Inflammatory myofibroblastic tumor	7	2	28.6
Bone	C40, C41	Bone	Chondrosarcoma	19	1	5.3
Bone	C40, C41	Bone	Chordoma	14	2	14.3
Bone	C40, C41	Bone	Osteosarcoma	43	5	11.6
Bone	C40, C41	Bone	Ewing sarcoma	263	12	4.6
Skin	C44	Skin	Basal cell carcinoma	12	5	41.7
Skin	C44	Skin	Merkel cell carcinoma	63	15	23.8
Skin	C44	Skin	Cutaneous squamous cell carcinoma	123	101	82.1
Lung	C39	Lung	Spindle cell carcinoma of the lung	3	2	66.7
Lung	C39	Lung	Lymphoepithelioma-like carcinoma of the lung	1	1	100.0
Lung	C39	Lung	Sarcomatoid carcinoma of the lung	15	7	46.7
Breast	C50	Breast	Adenoid cystic breast cancer	14	6	42.9
Breast	C50	Breast	Breast invasive mixed mucinous carcinoma	43	4	9.3
Urinary	C67	Bladder	Plasmacytoid/signet ring cell bladder carcinoma	6	5	83.3
Urinary	C67	Bladder	Sarcomatoid carcinoma of the urinary bladder	2	1	50.0
Urinary	C67	Bladder	Small cell bladder cancer	2	1	50.0
Urinary	C64	Kidney	Renal non-clear cell carcinoma	146	8	5.5
Others	C45, C48	Pleura, Peritonea	Pleural mesothelioma	75	1	1.3
Others	C37	Thymus	Thymic carcinoma	10	4	40.0
Others	C06	Head and neck	Adenoid cystic carcinoma	323	147	45.5
Others	C06	Head and neck	Salivary adenocarcinoma	4	1	25.0
Others	C06	Head and neck	Salivary duct carcinoma	19	16	84.2
Others	C06	Head and neck	Epithelial-myoepithelial carcinoma	3	1	33.3
Others	C80, C76	Unknown	Cancer of unknown primary	149	60	40.3
Summary				4,901	1,006	20.5

*#Each sample may have more than one targetable mutations, thus the prevalence may be over 100*.

### Mutation Profiling of Chinese Patients With Rare Tumors

We recruited a second, independent patient cohort from another pan-China database, Geneplus. One thousand three hundred and twelve patients (1312) with rare tumors were included for the study. The clinicopathological characteristics of all the patients are summarized in Table 4. The median age was 56, and 53.4% (700/1312) of the cohort were male. Ninety two percent (92.1%, 1209/1312) of the patients were at stage IV, and 58.6% (769/1312) of the patient were systemic treatment-naïve while 36% (472/1312) had been systemically treated. Tumor tissue was available for genetic analysis in 770 of these patients, while 469, 27, 16, 1, and 1 patient, respectively, had ctDNA, pleural effusion, peritoneal effusion, pericardial effusion, and cerebrospinal fluid (CSF) available as an alternative.

**Table 4:** Clinicopathological characteristics of patients.

**Characteristic**	**Pts. (*N* = 1,312)**
**Clinicopathological characteristics of patients**
Age, years	
median	56
Range	2–97
Gender	
Female	612
Male	700
Clinical stage	
I	5
II	31
III	34
IV	1,209
NA	33
Previous treatment	
Surgery	71
No systemic treatment	769
Systemically treated	472
Specimen	
Tumor tissue	770
ctDNA	496
Pleural effusion	27
Peritoneal effusion	16
Pericardial effusion	1
CSF	1

These 1,312 cases included 67 tumor subtypes out of our defined rare tumor types, with cancer of unknown primary, gastrointestinal stromal tumor, gallbladder cancer, intrahepatic cholangiocarcinoma, and sarcomatoid carcinoma of the lung as the top 5 tumors including 410, 107, 72, 70, and 51 patients, respectively.

Within these 1,312 samples, a total of 7,998 alterations were identified in 712 genes (5,924 base substitutions, 1,206 gene amplifications or deletions, 840 short indels, and 28 gene rearrangements) for a mean of 4 alterations per tumor (Supplementary Table 6). Total 701 targetable mutations were identified in the 1,312 samples, with *EGFR, KIT, CDKN2A, PIK3CA, PTEN, NF1, ERBB2, BRAF, BRCA2*, and *FGFR1/2/3* as the top 10 genes with targetable mutations identified in 266, 149, 119, 119, 112, 75, 66, 33, 33, 27, and 27 samples, respectively. Of the 1312 patients, 478 patients had at least 1 targetable mutation (Table 5 and Supplementary Table 7).

**Table 5:** Prevalence of targetable mutations in rare tumor samples from Chinese patients.

**System**	**ICD**	**Site**	**Tumors, including but not restricted to**	**Number of cases**	**Cases with targetable gene alterations**	**Prevalence of targetable gene alterations #(%)**	**Tissue**	**Number of patients with targetable gene alterations**
Digestive	C24	Biliary tract	Perihilar cholangiocarcinoma	30	12	40.0	17	10
Digestive	C24	Biliary tract	Extrahepatic cholangiocarcinoma	4	3	75.0	4	3
Digestive	C24	Biliary tract	Intrahepatic cholangiocarcinoma	70	24	34.3	34	13
Digestive	C23	Gallbladder	Gallbladder cancer	72	26	36.1	39	22
Digestive	C17	Small bowel	Small bowel well-differentiated neuroendocrine tumor	2	0	0.0	2	0
Digestive	C17	Small bowel	Duodenal adenocarcinoma	38	12	31.6	22	8
Digestive	C17	Small bowel	Small intestinal carcinoma	32	25	78.1	18	14
Endocrine	C74	Adrenal	Adrenocortical carcinoma	10	0	0.0	7	0
Endocrine	C75	Pituitary	Pituitary carcinoma	1	1	100.0	0	1
Endocrine	C73	Thyroid	Medullary thyroid cancer	15	5	33.3	13	5
Neural system	C72, C70	Brain	Anaplastic astrocytoma	6	9	150.0	6	6
Neural system	C72, C70	Brain	Anaplastic oligodendroglioma	2	1	50.0	2	1
Neural system	C72, C70	Brain	Anaplastic oligoastrocytoma	1	0	0.0	0	0
Neural system	C72, C70	Brain	Glioblastoma	32	74	231.3	30	26
Neural system	C72, C70	Brain	Astrocytoma	33	13	39.4	32	14
Neural system	C72, C70	Brain	Oligodendroglioma	6	0	0.0	6	0
Neural system	C72, C70	Brain	Oligoastrocytoma	2	0	0.0	0	0
Neural system	C72, C70	Brain	High-grade glioma(NOS)	5	6	120.0	5	4
Neural system	C72, C70	Brain	Primitive neuroectodermal tumor	7	1	14.3	4	1
Neural system	C72, C70	Brain	Medulloblastoma	2	0	0.0	0	0
Neural system	C72, C70	Brain	Anaplastic meningioma	1	1	100.0	0	1
Neural system	C72, C70	Brain	Meningioma	10	6	60.0	5	6
Neural system	C72, C70	Brain	Rhabdoid meningioma	1	2	200.0	1	1
Neural system	C72, C70	Brain	Malignant teratoma	1	0	0.0	0	0
Neural system	C72, C70	Brain	Embryonal carcinoma	2	0	0.0	1	0
Neural system	C72, C70	Brain	Choriocarcinoma	1	0	0.0	1	0
Neural system	C72, C70	Brain	Ependymoma	3	1	33.3	3	1
Neural system	C72, C70	Brain	Anaplastic ependymoma	4	2	50.0	4	1
Neural system	C47	Peripheral Nerve	Malignant peripheral nerve sheath tumor	5	5	100.0	4	2
Reproductive	C60	Penile	Penile squamous cell carcinoma	6	7	116.7	3	4
Reproductive	C52, C51	Vulva/vagina	Squamous cell carcinoma of the vulva/vagina	8	2	25.0	3	2
Reproductive	C52, C51	Vulva/vagina	Vaginal adenocarcinoma	2	0	0.0	0	0
Reproductive	C56	Ovary	Dysgerminoma	1	0	0.0	1	0
Reproductive	C56	Ovary/vulva/vagina/brain/testis,	Embryonal carcinoma	2	0	0.0	1	0
Soft tissue	C49	Soft tissue	Desmoplastic small-round-cell tumor	1	0	0.0	0	0
Soft tissue	C49	Soft tissue	Rhabdomyosarcoma	16	6	37.5	9	6
Soft tissue	C49	Soft tissue	Synovial sarcoma	16	0	0.0	15	0
Soft tissue	C49	Soft tissue	Myofibroma	2	0	0.0	1	0
Soft tissue	C49	Soft tissue	Myxofibrosarcoma	3	3	100.0	0	1
Soft tissue	C49	Soft tissue	Leiomyosarcoma	48	11	22.9	34	9
Soft tissue	C49	Soft tissue	Alveolar soft part sarcoma	7	0	0.0	6	0
Soft tissue	C49	Soft tissue	Epithelioid sarcoma	5	0	0.0	0	0
Soft tissue	C49	Soft tissue	Epithelioid hemangioendothelioma	2	1	50.0	1	1
Soft tissue	C49	Soft tissue	Dendritic cell sarcoma	1	1	100.0	0	1
Soft tissue	C49	Soft tissue	Clear cell sarcoma	3	1	33.3	0	1
Soft tissue	C49	Soft tissue	Undifferentiated pleomorphic sarcoma/malignant fibrous histiocytoma/high-grade spindle cell sarcoma	12	3	25.0	7	2
Soft tissue	C49	Soft tissue	Gastrointestinal stromal tumor	107	113	105.6	82	79
Soft tissue	C49	Soft tissue	Fibrosarcoma	7	1	14.3	6	1
Soft tissue	C49	Soft tissue	Angiosarcoma	6	1	16.7	2	1
Soft tissue	C49	Soft tissue	Inflammatory myofibroblastic tumor	6	0	0.0	5	0
Soft tissue	C49	Soft tissue	Desmoid/aggressive fibromatosis	1	0	0.0	0	0
Soft tissue	C49	Soft tissue	Liposarcoma	19	1	5.3	14	1
Bone	C40, C41	Bone	Chondrosarcoma	6	2	33.3	6	1
Bone	C40, C41	Bone	Chordoma	2	0	0.0	1	0
Bone	C40, C41	Bone	Osteosarcoma	18	2	11.1	9	2
Skin	C44	Skin	Dermatofibrosarcoma protuberans	2	1	50.0	1	1
Skin	C44	Skin	Cutaneous squamous cell carcinoma	5	3	60.0	3	2
Skin	C44	Skin	Sebaceous carcinoma	1	0	0.0	1	0
Skin	C44	Skin	Sweat gland adenocarcinoma	2	3	150.0	0	2
Skin	C44	Skin	Sweat gland carcinoma/apocrine eccrine carcinoma	4	3	75.0	4	2
Lung	C39	Lung	Sarcomatoid carcinoma of the lung	51	28	54.9	28	20
Urinary	C64	Kidney	Renal non-clear cell carcinoma	49	20	40.8	21	14
Others	C45, C48	Pleura, peritonea	Pleural mesothelioma	21	3	14.3	12	3
Others	C45, C48	Pleura, peritonea	Pleuropulmonary blastoma	2	1	50.0	0	1
Others	C45, C48	Pleura, peritonea	Peritoneal mesothelioma	12	4	33.3	6	3
Others	C37	Thymus	Thymic carcinoma	48	15	31.3	25	12
Others	C80, C76	Unknown	Cancer of unknown primary	410	236	57.6	189	166
Summary				1,312	701	53.4	756	478

*#Each sample may have more than one targetable gene alterations, thus the prevalence may over 100*.

### Consistencies and Discrepancies Between the Two Cohorts of Rare Tumors

Between the cBioPortal cohort and our independent cohort, there were 41 overlapping subtypes (41/63, cBioPortal; 41/67, our cohort) and 22 (cBioPortal) or 25 (our cohort) subtypes unique to each cohort (Table 6, Supplementary Figure 1).

**Table 6:** Percentage of targetable mutation carrier in the two cohorts.

**Gene**	**Genomic alteration**	**Approved targeted therapies**	**cBioPortal (%)**	**Geneplus cohor (%)**
*ALK*	Fusion	**Crizotinib, Ceritinib, Alectinib**, Brigatinib	0.55	1.07
*ATM*	Substitution, truncation	**Olaparib**, Talazoparib, Rucaparib, Niraparib	1.53	1.83
*BRAF*	Substitution, fusion	Vemurafenib, Dabrafenib, **Regorafenib, Sorafenib**, Trametinib	0.67	1.91
*BRCA1*	Substitution, truncation	**Olaparib**, Talazoparib, Rucaparib, Niraparib	0.33	0.23
*BRCA2*	Substitution, truncation	**Olaparib**, Talazoparib, Rucaparib, Niraparib	0.55	1.91
*CDKN2A*	Loss, substitution, truncation	**Palbociclib**, Ribociclib, Abemaciclib	2.29	7.24
*EGFR*	Substitution	**Erlotinib, Afatinib, Gefitinib, Icotinib, Osimertinib**, Lapatinib, Dacomitinib	0.47	7.70
*ERBB2*	Amplification, substitution	**Trastuzumab, Lapatinib, Pyrotinib**, Pertuzumab, **Trastuzumab-DM1, Afatinib**	0.53	3.28
*FGFR1,2,3*	Substitution, amplification, fusion	Erdafitinib, **Pazopanib**, Ponatinib	1.33	1.91
*KIT*	Substitution	**Imatinib**	2.43	7.32
*MET*	Amplification	**Crizotinib**, Cabozantinib	0.18	1.60
*NF1*	Loss, truncation	**Temsirolimus, Everolimus**, Trametinib	0.67	4.34
*NTRK1,2,3*	Fusion	Larotrectinib	0.10	0.08
*PIK3CA*	Substitution, amplification	Alpelisib, **Temsirolimus, Everolimus**	5.39	6.86
*PTEN*	Loss, substitution, truncation	**Temsirolimus, Everolimus**	2.98	5.34
*RET*	Fusion/substitution	Cabozantinib, Ponatinib, Sorafenib, **Sunitinib**, Vandetanib, **Regorafenib**	0.37	0.61
*ROS1*	Fusion	**Crizotinib, Ceritinib**	0.04	0.23

*Bold: approved by NMPA*.

**Figure 1:**
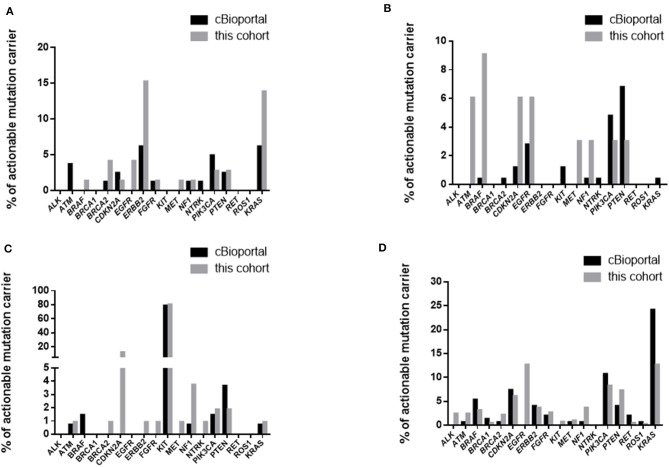
Comparison of targetable mutations in gallbladder cancer **(A)**, astrocytoma **(B)**, gastrointestinal stromal tumor **(C)**, and cancer of unknown primary **(D)**.

We first compared the overall prevalence of TGAs in these two cohorts. The prevalence of targetable mutations was significantly higher in our cohort compared with the data from cBioPortal (53.4 vs. 20.4%, *p* < 0.001) (Table 6). Specifically, mutations or amplifications of *BRAF, BRCA2, CDKN2A, EGFR, ERBB2, KIT, MET, NF1, ROS1* were 3 or more times more frequent in our cohort than in the cBioPortal cohort. Alterations of *BRCA1, NTRK* fusion were slightly more common in the cBioPortal cohort. When restricting analysis to the 41 overlapping subtypes, the difference of targetable mutations was still significant (54.1 vs. 26.1%, *p* < 0.001). We further focused on 4 rare tumors (gallbladder cancer, astrocytoma, gastrointestinal stromal tumor, and cancer of unknown primary) with more than 30 cases in both cohorts. We found the overall incidence rate of targetable mutations was higher in our cohort (Supplementary Table 8). For gallbladder cancer, *ERBB2* and *BRCA2* mutations were significantly more frequent in our cohort, while *ATM* mutation was enriched in the cBioPortal cohort (Figure 1A) (23). For astrocytoma, *BRAF, ATM, CDKN2A*, and *EGFR* mutations/amplifications were highly enriched in our cohort (Figure 1B). For gastrointestinal stromal tumor, the prevalence of the *KIT* mutation was similar between the two groups, but our cohort had a significantly higher prevalence of *CDKN2A* and *NF1* (Figure 1C). For cancer of unknown primary, *EGFR* mutation and *ALK* fusion were highly enriched in our cohort, which indicate that those tumors might originate from lung (Figure 1D).

## Discussion

This study focused on rare tumors in China and proposed a novel definition of rare tumors customized for China by jointly considering frequency and clinical characteristics to addresses the disparate requirements of clinical decision-making, clinical research, drug development, and health care services. Applying this new definition, a comprehensive list of rare tumors was explored for genetic biomarkers of response to targeted therapy both in the worldwide cBioPortal database and a mainland China-specific patient cohort mainly to explore potential novel treatment indications for those rare tumors in China. Results show that targetable gene alterations are frequently present in rare tumors, and that these mutations are enriched in Chinese population as compared to the general global population.

Most importantly, a definition of rare tumors in China was proposed for the first time based on the epidemiology data and availability of standard treatment in China. An incidence of ≤2.5/100,000 per year as a cut off value for rare tumor in China is novel and it is rigorous compared with those of the USA and Europe which is 15/100,000 and 6/100,000 respectively. The disparity should be mainly attributed to the facts that China has a larger population base, and a different epidemiological distribution for most types of tumors compared to western countries. We believe any threshold for rarity is artificial and should be considered as just indicative. We should always be aware that an incidence threshold rate as a line for rareness should be used with flexibility. The most important purpose of proposing the definition is to increase the attention from clinical practitioners and government personnel of China, as well as drug investigators all over the world, to promote the development of novel drugs and strategies for those rare tumors without consensus and guidelines for effective treatment in China, and finally to improve the outcome of rare tumor patients.

After applying our rare tumor criteria to patient data, we discovered the overall prevalence of TGAs in Chinese rare tumor patients' cohort was much higher than that of the cBioPortal cohort. We restricted our analysis of TGAs to genes having Level 1-4 evidence of being a cancer gene according to the OncoKB knowledge database. Using this framework, we identified mutations of *ALK, ATM, BRAF, BRCA1, BRCA2, CDKN2A, EGFR, ERBB2, FGFR1,2,3, KIT, MET, NF1, NTRK1,2,3, PIK3CA, PTEN, RET*, and *ROS1* within our cohort. The cumulative prevalence of TGAs was significantly higher in Chinese cohort (53.43%) compared with general population worldwide (26.1%). This indicates that there might be higher possibilities those patients could benefit from targeted therapies. The underlying causes for the disparities in mutation prevalence were complicated as the two cohorts had significantly different compositions of tumor subtypes, as well as different numbers of patients in each subtype. The overall difference between the two cohorts was still significant (*p* < 0.001) if we only studied the shared 41 subtypes of rare tumor. This phenomenon is in agreement with the data showing that *EGFR* mutation rate in Asian NSCLC patients is higher than that of Caucasian patients. Our findings indicate that the classification of “rare tumor” is heterogeneous by ethnicity.

We also found that most common TGAs in both cohorts are actionable with available drugs. The top 5 targetable mutations found in Chinese patients cohort were *EGFR, KIT, CDKN2A, PIK3CA*, and *PTEN*; and in the cBioPortal cohort were *PIK3CA, PTEN, KIT, CDKN2A*, and *ATM*. Regarding the 4 shared targetable mutations, there is at least one targeted drug for each mutation (imatinib for *KIT*, palbociclib for *CDKN2A*, temsirolimus and everolimus for *PIK3CA* and *PTEN*) currently available in China (Table 6). This suggests that we have available effective treatment options for some rare tumor patients.

Finally, our data indicate that samples for genetic profiling of rare tumor are still inadequate. There are only 10.5% (4901/46566) tumor samples from rare tumors in cBioPortal database. Moreover, 52 out of 141 (36.9%) subtypes of rare tumors did not have genetic data available in cBioPortal or in our cohort (Supplementary Table 9). For most subtypes with data, the median number of samples was 19 in cBioPortal and 5 in our cohort. Considering the high prevalence of TGAs in the rare tumor population and the largely unmet medical needs of those patients, more attention and efforts should be applied in this field in the near future.

## Conclusions

We defined rare tumor in China as ICD-specified tumors with incidence ≤2.5/100,000 per year in China, and subtypes of non-rare ICD-specified tumors with incidence ≤2.5/100,000 per year in China, and cancers of unknown primary. Genomic profiling of rare tumors matching this definition from cBioPortal and a Chinese cohort drawn from the Geneplus database demonstrated a substantial prevalence of targetable genomic alterations in these tumors, which was even higher in Chinese rare tumor patient population than in the general population. All of the above facilitates future drug investigations and treatment improvement for rare tumors.

## Data Availability Statement

The datasets used and/or analyzed during the current study are available from the corresponding author on reasonable request.

## Ethics Statement

This study was approved by the ethics committees of the National Cancer Center/Cancer Hospital, Chinese Academy of Medical Sciences and Peking Union Medical College (NCC2019C-222). All patients signed written informed consent for further scientific analysis of genetic data.

## Author Contributions

NL and XY conceived the study. SW and RC processed data, performed data analysis. YT, YY, YF, HH, DW, HF, YB, CS, AY, QF, and DG. contributed to data collection, generation of tumor list and scientific insights. SW and RC wrote the manuscript. SW, NL, and XY revised the manuscript.

## Conflict of Interest

The authors declare that the research was conducted in the absence of any commercial or financial relationships that could be construed as a potential conflict of interest.

## Abbreviations

NGS: Next generation sequencing
TGAs: targetable genomic alterations
cBioPortal: CBio Cancer Genomics Portal
ESMO: European Society for Medical Oncology
NCI: National Cancer Institute
FDA: Food and Drug Administration
ICD: International Classification of Diseases
CNKI: China National Knowledge Infrastructure
CUP: cancers of unknown primary
MSK: Memorial Sloan Kettering Cancer Center
SNV: Single nucleotide variants
InDels: insertions and deletions
CSF: cerebrospinal fluid.
