# Supplementary figures and images for: Comprehensive Genomic Profiling of Rare Tumors: Routes to Targeted Therapies
Source: Front Oncol. 2020 Apr 21;10:536. doi: 10.3389/fonc.2020.00536 (PMC7186305; doi:10.3389/fonc.2020.00536)

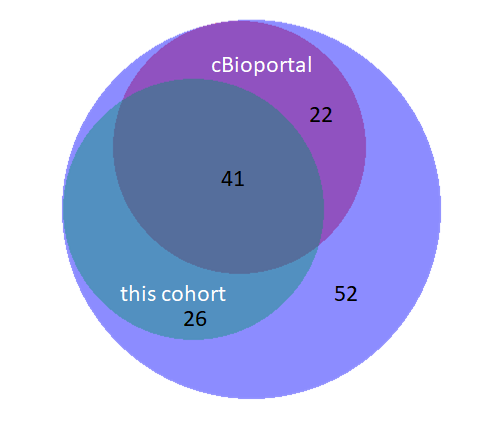


**Supplementary figure 1**. Venny figure of subtypes of rare tumors included in the two cohort.

Supplement: Supplementary file 10 [file Table_10.docx]
